# Organic Nitrogen-Driven Stimulation of Arbuscular Mycorrhizal Fungal Hyphae Correlates with Abundance of Ammonia Oxidizers

**DOI:** 10.3389/fmicb.2016.00711

**Published:** 2016-05-12

**Authors:** Petra Bukovská, Milan Gryndler, Hana Gryndlerová, David Püschel, Jan Jansa

**Affiliations:** Laboratory of Fungal Biology, Institute of Microbiology, Czech Academy of SciencesPrague, Czech Republic

**Keywords:** soil heterogeneity, organic amendments, arbuscular mycorrhizal (AM) fungi, soil hyphae, microbial communities, 454-amplicon sequencing, quantitative real-time PCR (qPCR), ammonia oxidizers

## Abstract

Large fraction of mineral nutrients in natural soil environments is recycled from complex and heterogeneously distributed organic sources. These sources are explored by both roots and associated mycorrhizal fungi. However, the mechanisms behind the responses of arbuscular mycorrhizal (AM) hyphal networks to soil organic patches of different qualities remain little understood. Therefore, we conducted a multiple-choice experiment examining hyphal responses to different soil patches within the root-free zone by two AM fungal species (*Rhizophagus irregularis* and *Claroideoglomus claroideum*) associated with *Medicago truncatula*, a legume forming nitrogen-fixing root nodules. Hyphal colonization of the patches was assessed microscopically and by quantitative real-time PCR (qPCR) using AM taxon-specific markers, and the prokaryotic and fungal communities in the patches (pooled per organic amendment treatment) were profiled by 454-amplicon sequencing. Specific qPCR markers were then designed and used to quantify the abundance of prokaryotic taxa showing the strongest correlation with the pattern of AM hyphal proliferation in the organic patches as per the 454-sequencing. The hyphal density of both AM fungi increased due to nitrogen (N)-containing organic amendments (i.e., chitin, DNA, albumin, and clover biomass), while no responses as compared to the non-amended soil patch were recorded for cellulose, phytate, or inorganic phosphate amendments. Abundances of several prokaryotes, including *Nitrosospira* sp. (an ammonium oxidizer) and an unknown prokaryote with affiliation to *Acanthamoeba* endosymbiont, which were frequently recorded in the 454-sequencing profiles, correlated positively with the hyphal responses of *R. irregularis* to the soil amendments. Strong correlation between abundance of these two prokaryotes and the hyphal responses to organic soil amendments by both AM fungi was then confirmed by qPCR analyses using all individual replicate patch samples. Further research is warranted to ascertain the causality of these correlations and particularly which direct roles (if any) do these prokaryotes play in the observed AM hyphal responses to organic N amendment, organic N utilization by the AM fungus and its (N-unlimited) host plant. Further, possible trophic dependencies between the different players in the AM hyphosphere needs to be elucidated upon decomposing the organic N sources.

## Introduction

The physical arrangement of soil particles, aggregates, and pores; root and microbial growth; and burrowing activities of animals, together with external inputs of such particulate organic residues as plant litter, animal excreta, and dead bodies, all create a soil environment highly heterogeneous in both space and time and at a range of scales (Facelli and Facelli, 2002; Watt et al., 2006). Adaptations of roots to heterogeneously distributed organic and inorganic nutrients in soil have been studied for decades (Robson et al., 1992; Robinson, 1996; Hodge, 2004). For most plants, however, the root is not the only – and possibly not even the main – organ for primary acquisition of such poorly mobile nutrients as phosphorus (P) from the soil solution. This function is often fulfilled by the plants’ mycorrhizal symbionts. Arbuscular mycorrhizal (AM) fungi, belonging to the Glomeromycota, establish intimate relationships with more than half of currently described plant species (van der Heijden et al., 2015). The nutrients taken up by AM fungal hyphae from the soil solution are then passed on to the host plants at the root–mycorrhizal interface in the root cortex (Fitter, 1991; Smith et al., 2004). The importance of AM symbiosis for P acquisition by many plant species is firmly established (Cox and Tinker, 1976; Jakobsen et al., 1992), whereas its role in plant nitrogen (N) acquisition, although repeatedly demonstrated (Mäder et al., 2000; Hodge et al., 2001; Fellbaum et al., 2012), is broadly accepted as being lower than that in plant P acquisition (Johnson et al., 2015).

How AM hyphal networks respond to heterogeneously distributed soil resources and what consequences this has for their own as well as for host plant nutrition is much less understood than are root responses. Previously, we and others have shown that at least some AM fungal species establish denser hyphal networks in root-free patches as compared to the rooting zones (Jansa et al., 2003; Thonar et al., 2011; Zheng et al., 2015) and that root responses to heterogeneously distributed soil nutrients could be negated through the establishment of AM symbiosis (Felderer et al., 2013). This could be caused by positive hyphal developmental response to patches with greater availability of mineral nutrients (Li et al., 1991; Zheng et al., 2015), to specific N forms within the patches (Bago et al., 2004), or to variation in such other soil physicochemical properties as clay or organic matter contents (Jansa et al., 2003). Indeed, significant research efforts have been dedicated in the past to deciphering the response of AM fungal networks to soil organic matter. It has previously been shown that extracted soil organic matter, dried plant biomass, dried baker’s yeast, and bovine serum albumin specifically stimulated the development of AM fungal networks in root-free patches and that starch and pure cellulose have depressed it (St. John et al., 1983; Joner and Jakobsen, 1995; Ravnskov et al., 1999; Gavito and Olsson, 2003; Leigh et al., 2009). Other experiments, while not specifically distinguishing between root and root-free zones, have recorded significant stimulation of the development of AM extraradical hyphae, root colonization, and sporulation by the addition of crab-shell chitin to the rhizosphere (Gryndler et al., 2003). However, the large biological variation in mycorrhizal experiments and the use of different soils, model plants, and fungal species in the diverse studies testing the various organic amendments **one by one** preclude direct comparisons as to the effects exerted by different compounds. Surprisingly, to the best of our knowledge, a multidimensional model used previously to examine preferences of AM hyphal networks to colonize spatially discrete patches having different mineral fertilizer amendments in a monoxenic cultivation system (Bago et al., 2004) as well as in planted or unplanted soil patches (Gavito and Olsson, 2008) has not yet been employed to study the effect of different qualities of soil organic patches within the reach of a single AM fungal colony. Since the host plant in most previous experiments has been N-limited (e.g., Leigh et al., 2009), AM-induced mineralization of organic N in those experiments might have been driven by the high N demand of both the host plant and the AM fungi. What would be the choice of AM fungi for certain organic patches if the host plant was not N-, but P-limited, has not been sufficiently addressed as yet.

It had previously been postulated that at least part of the response of AM fungal hyphae to soil organic amendments is caused and/or modulated by other soil microbes because the saprotrophic potential of AM fungi is thought to be low (Joner et al., 2000; Gryndler et al., 2003; Leigh et al., 2009). It could be that those microbes live solely upon (derive their energy from) the organic materials, release mineral nutrients (N and/or P) from them, and produce other (secondary) metabolites. In such case, the nutrients or the other metabolites could then be involved in the AM hyphal response. On the other hand, the microbes might also associate directly with the AM hyphal surfaces (Toljander et al., 2006; Jansa and Gryndler, 2010), partially or fully derive their carbon/energy from the mycorrhizal hyphae (Toljander et al., 2007; Drigo et al., 2010), and carry on degradation of the soil organic materials in order to release the mineral nutrients either for themselves or possibly for their fungal hosts (Jansa et al., 2013).

Because knowledge is so fragmented as to the responses of AM fungal hyphae to different soil organic amendments containing or not containing such mineral nutrients as P and/or N, we carried out a **multiple-choice experiment** whereby the development of hyphal networks was quantified within a number of spatially discrete patches buried in the root-free zone of each pot. The goal was to directly compare the **responses of a single AM hyphal network** to different soil organic amendments, containing either N or P or both or none of these nutrients. Using the available high-throughput sequencing technology and quantitative real-time PCR (qPCR) we aimed at identification of a possible common denominator of the AM fungal response to the amendments within the soil microbe (prokaryotic and fungal) communities. Because the host plant (*Medicago truncatula*) requirements for N were likely saturated via atmospheric N fixation and because it was grown under P-limiting conditions, we hypothesized (based on the resource limitation theory, Johnson et al., 2015) that the AM hyphae would preferentially colonize the P-containing organic materials due to higher demand for P than N by the host plant. This hypothesis is in line with some earlier observations that AM hyphae could contribute a large share of plant P uptake from organic P sources accessible only to the hyphae (Tarafdar and Marschner, 1994; Feng et al., 2003; Zhang et al., 2014).

## Materials and Methods

### Pot Setup and Patch Enrichment

The experiment was conducted in 10 l plastic pots lined with a polyamide mesh fabric (mesh size 1 mm) at the bottom, filled with a substrate consisting of autoclaved quartz sand (grain size < 3 mm), autoclaved zeolite MPZ 1-2.5 (Zeopol^1^, grain size 1–2.5 mm) and γ-irradiated (>25 kGy) soil from Litoměřice, Czech Republic (N 50°31′54.53″, E 14°06′7.10″; pH_H2O_ 7.88; 42% clay, 40% sand; total P [i.e., P extractable with hot 14 M HNO_3_ after incineration at 550°C] 797 mg kg^-1^; water-extractable P 3.3 mg kg^-1^; total organic C 2.26%; total N 0.13%) mixed in a 9:9:2 ratio (v:v:v). The properties of the potting substrate (hereinafter referred to as “soil”) were as follow: pH_H2O_ 8.9, total P 46.5 mg kg^-1^, water extractable P 2.6 mg kg^-1^, total organic C 0.22%, total N 0.013%. The entire volume of the soil was inoculated with non-mycorrhizal (mock) inoculum (1%, v:v), consisting of the substrate and root fragments from a previous pot culture planted with leek and grown in a greenhouse for 10 months without inoculation by any AM fungus. This was done so as to introduce a standardized microbial community into the previously sterile soil. The plant (central) compartments (500 ml volume, manufactured from a 40 μm polyamide mesh; Silk & Progress, Brněnec, Czech Republic, Supplementary Figure S1) were inoculated with either *Rhizophagus irregularis* BEG 158 or *Claroideoglomus claroideum* BEG 155 (2%, v:v), supplied as substrate and root fragments from a previous pot culture containing the respective fungal isolate, planted with leek, and grown in a greenhouse for 10 months prior to the experiment described here. Eight replicate pots per fungal treatment were established and their positions in the glasshouse completely randomized.

Using pot-based inoculum of AM fungi (and the relevant mock-inoculum containing similar microbial community as the AM fungal inoculum) was necessary because the AM fungi are obligate biotrophs that absolutely require presence of host plant roots for their growth, and the production of AM fungal inoculum in root-organ cultures is currently limited to a single AM fungal genus (*Rhizophagus*). Using open pot cultures is the standard way to produce AM inoculum for experimental and biotechnology purposes (Calvet et al., 2013) and using the appropriate mock inoculum is necessary to control for other potentially confounding biotic effects (Wagg et al., 2014).

Plant compartments were surrounded by a set of eight patches, filled with the same soil as above and amended or not amended with different organic materials or orthophosphate (in form of Na_2_HPO_4_.12H_2_O). The patches (Supplementary Figure S1) were made of PVC tubing (diameter 3.6 cm, length 3 cm), with the open sides covered by 100 μm mesh (Silk & Progress). The patches were arranged 3 cm from the plant compartment, 4 cm above its bottom, and clockwise in the sequence given in **Table 1**. The organic compounds were purchased from Sigma-Aldrich (St. Louis, MO, USA); chitin (from crab shells) was milled to pass through a 0.5 mm sieve. Crystalline sodium dihydrogen phosphate was provided by a local chemical supplier (P-lab, Prague, Czech Republic) and clover (*Trifolium repens*) aboveground biomass was collected at a nearby grassland, dried at 65°C, then milled to pass through a 0.5 mm sieve. Calculated values of C, N, and P concentrations in the pure chemicals and analyses of C, N, and P concentrations in the clover biomass were used to calculate the levels of soil amendments as follows:

**Table 1:** List of compounds and their amounts used to create amended soil patches showing the assumed and measured levels of carbon (C), nitrogen (N), and phosphorus (P) inputs per unit volume of soil.

Amendment	Origin/supplier/cat. number	Amount of amendment added to soil (g l^-1^)	Assumed input (g l^-1^)			Measured input (g l^-1^)		
			C	N	P	C	N	P
Cellulose	Sigma S3504	2	0.887	0	0	0.802	nd	nd
Chitin	Sigma C4666	2	0.940	0.138	0	0.828	0.131	0.002
Albumin	Sigma A7906	1	0.449	0.138	0	0.491	0.160	nd
DNA	Sigma 31149	0.85	0.308	0.138	0.085	0.275	0.124	0.069
Phytate	Sigma P8810	0.31	0.033	0	0.085	0.026	nd	0.062
Orthophosphate	P-lab H08102	0.98	0	0	0.085	<0.001	nd	0.084
Clover biomass	Collected from a grassland	2.18	0.917	0.065	0.003	0.897	0.085	0.007
Control	na	none	na	na	na	na	na	na

*Assumed inputs were based on published elemental composition of pure compounds and preliminary analyses of clover biomass and used to prepare soil patches. na, not applicable; nd, not detected.*

The levels of cellulose and chitin amendments in the patches were arbitrarily set to 2 g per liter of soil based on previous experiments showing stimulatory effects at these levels of soil amendment with chitin on mycorrhizal colonization of roots and hyphal densities in the soil (Gryndler et al., 2003). The levels of albumin and DNA amendments were adjusted so that the N inputs from these compounds would be the same as the N inputs in the chitin treatment (**Table 1**). The levels of phytate and phosphate amendments were then adjusted so that they would match the P input of the DNA amendment (**Table 1**). The level of soil amendment with clover biomass was adjusted so that it would supply the same amount of C as the chitin, whereas the N inputs would be about half that of the chitin treatment (**Table 1**).

The plant compartments were planted with five pre-germinated *M. truncatula* (cultivar J5) seeds each at the beginning of the experiment (hereinafter referred to as “sowing”) and added with 10^10^ cells of *Sinorhizobium meliloti* strain 1021, cultivated in the tryptone yeast liquid medium (Somasegaran and Hoben, 1994) on a shaker at 24°C for 3 days. The pots were kept in the greenhouse of the Institute of Microbiology, Prague during the summer months (from 24 July until 27 September 2012), with temperature ranging between 18 and 36°C and the day length extended to 16 h with supplemental lighting (halogen lamps providing a minimum photosynthetic flux density of 200 μmol m^-2^ s^-1^). Plants were thinned to three individuals per pot during the third week of growth and fertilized from the fourth week on with a modified P2N3 white mineral nutrient solution (Gryndler et al., 1992) containing 20% of the previously described P concentration. Each pot received 25 ml of the nutrient solution each week between the fourth and sixth week of growth and 50 ml each week thereafter, with the nutrient solution applied only to the plant compartments. Each pot thus received 7.9 mg N and 0.06 mg P as soluble mineral fertilizer during the first 6 weeks of growth (until the first harvest), or 18.2 mg N and 0.14 mg P until the second harvest. Pots were watered daily with deionized water to maintain moisture at approximately 75% of the soil’s water holding capacity.

### Harvest and Plant Analyses

Four replicate pots of each AM fungal treatment were harvested 6 weeks after sowing and the other four replicates per fungal treatment were harvested 9 weeks after sowing. At harvest, the shoots were cut at soil level, then dried at 65°C for 7 days and weighed. Soil (between 20 and 30 g) was sampled from all patches, from the root-free volume between the patches, and from the plant compartments, then frozen at -20°C. Roots were washed from the plant compartments, weighed fresh, and then cut into 1 cm fragments, homogenized, and divided into three aliquots. One aliquot was kept in 50% ethanol for staining, one was frozen at -20°C, and one was weighed fresh, dried at 65°C for 7 days, and weighed dry. Knowing the fresh-to-dry biomass ratio of the root aliquot processed by drying, the entire root dry biomass per pot was calculated using the fresh biomass values of the entire root system.

Phosphorus concentration in the dried plant biomass samples was determined using 100 mg dry biomass aliquots, incinerated at 550°C for 12 h, the ashes dissolved in concentrated (14 M) HNO_3_, heated briefly to 200°C, brought up to 50 ml with ultrapure water, and the P concentration in the extracts measured spectrophotometrically using Malachite Green according to Ohno and Zibilske (1991). N concentration and isotopic composition in the plant samples were determined using an elemental analyzer (Flash EA 2000, ThermoFisher Scientific, Waltham, MA, USA) coupled with a Delta V mass spectrometer (ThermoFisher Scientific) while processing 2 mg milled biomass samples wrapped in tin capsules.

The extent of root length colonized by AM fungal structures was determined after Trypan blue staining following the standard protocol (Koske and Gemma, 1989) and using the magnified intersection method (McGonigle et al., 1990) while scoring 100 root intersections per sample.

Molecular quantification of the AM fungal development of the roots was carried out by qPCR using the **clar** (Thonar et al., 2012) and **mt5** (Couillerot et al., 2013) markers (primers and fluorescent probes) for *Claroideoglomus* and *Rhizophagus*, respectively, on DNA samples extracted from frozen roots using a Plant DNeasy Mini Kit (Qiagen, Venlo, Netherlands) and adding 2 × 10^10^ copies of internal DNA standard (as described in Thonar et al., 2012) to each sample before DNA extraction. The concentration of internal DNA standard in the extracts was measured with a specific qPCR marker described previously (Thonar et al., 2012) and used to correct the quantification of AM fungal DNA concentration in the samples for unspecific losses during extraction. qPCR analyses were carried out in 20 μl format using a StepOnePlus Real-Time PCR System (Life Technologies, Carlsbad, CA, USA) and 5× HOT FIREPol Probe qPCR Mix Plus-ROX (Solis BioDyne, Tartu, Estonia). Two microliters of template were added per reaction. The working concentration of each of the primers was 500 nM and that of the hydrolysis probe was 125 nM. The results of the analyses were expressed as number of rRNA gene copies or amount of AM fungal DNA per unit dry weight of roots, using PCR amplicon or pure fungal DNA for calibration, respectively, as described previously (Thonar et al., 2012).

### Analyses of AM Fungal Networks and Microbial Communities

The development of AM fungal networks in the soil was quantified following two independent approaches:

(a) Microscopy. Frozen soil samples were thawed, 10 g of the moist sample was washed through stacked 315 μm and 40 μm sieves, and the material collected in the finer (lower) sieve was homogenized in a blender for 10 s at high speed. The sample was then swirled with 500 ml water on a magnetic stirrer and five subsamples, 5 ml each, were taken at 10 s intervals after switching off the stirrer. These subsamples were combined and washed through a nitrocellulose membrane filter with imprinted grid (Millipore RAWG 1.2 μm, 25 mm diameter, EMD Millipore, Billerica, MA, USA), stained with Trypan blue (0.1% in 1% lactic acid) for 5 min, and AM hyphal intersections with the grid scored under a compound microscope at 200× magnification. Conversion to hyphal length density (i.e., hyphal length per unit dry weight of the soil) was carried out as described previously (Sylvia and Norris, 1992) using soil humidity estimates determined by weighing aliquots of the soil samples before and after drying at 65°C for 5 days.
(b) Quantitative Real-Time PCR: DNA was extracted from the frozen soil samples (500–800 mg fresh weight) using a NucleoSpin Soil kit (Macherey-Nagel, Düren, Germany) according to the manufacturer’s instructions and while spiking each sample with 2 × 10^10^ copies of the internal DNA standard before extraction. DNA concentration for the respective AM fungal taxon per unit dry weight of soil was determined by the specific qPCR markers and while correcting for internal standard losses and soil humidity as described above.

Based on the responses of AM hyphal networks to soil amendments, we selected all the different kinds of soil patches (as in **Table 1**) from the *Rhizophagus*-inoculated pots and the patches enriched with chitin, albumin, and clover biomass, as well as the control (non-amended) patches from the *Claroideoglomus*-inoculated pots, all from the second harvest, for analyses of eubacterial and fungal communities by 454-sequencing. To this end, equal amounts of DNA extracts from the four replicate patches of the same kind in each fungal treatment were pooled and separately amplified with ITS1F/ITS4 (White et al., 1990) and eub530F/1100aR (Baldrian et al., 2012) primer pairs using the PPP master mix (Top-Bio, Prague, Czech Republic), containing 1.5 Unit of Taq polymerase and supplemented with 0.05 Unit Pfu proof-reading polymerase (ThermoFisher Scientific) per 25 μl reaction. Each amplification was performed in analytical triplicates, which were then pooled and the amplicons purified with QIAquick PCR purification kit (Qiagen). Equimolar mixtures of bacterial and eukaryotic amplicons obtained from each of the 12 samples (eight from *Rhizophagus*-inoculated pots and four from *Claroideoglomus*-inoculated pots) were then fused with the multiplex identifier adaptors using a Lib-L kit (Roche, Basel, Switzerland) following the manufacturer’s recommendations, with the short fragments subsequently removed by Agencourt AMPure XP PCR purification kit (Beckman Coulter, Brea, CA, USA). Equimolar mixtures of fused amplicons from all samples were subjected to emulsion PCR using the GS Junior Titanium emPCR Kit and sequencing on the GS Junior platform (Roche).

Sequencing data were treated by SEED software (Větrovský and Baldrian, 2013): after removing low-quality sequences (reads), removing reads shorter than 400 bp, denoising, and excluding chimeras, the remaining reads were twice clustered at the similarity level of 95% (eubacteria) or 97% (fungi). For each primer pair, sequencing of multiplex identifier-marked library resulted in a data set containing sequence reads with forward or reverse orientation. Thus, each operational taxonomic unit (OTU) was physically detected as two different clusters of sequences with opposite orientation. For subsequent statistical analyses, all clusters were treated as separate entities. The sequences have been deposited in the open access Sequence Read Archive^2^ under study accession number SRP051101.

Based on the results of 454-sequencing, we designed two novel qPCR markers (specific primers + matching hydrolysis probes, see Supplementary Table S1 for details) for *Nitrosospira* sp. and for a bacterium showing similarity to *Acanthamoeba* endosymbiont (Candidatus *Caedibacter acanthamoebae*), both of which showed significant and positive correlation with the *R. irregularis* developmental response to soil amendments. These markers were then used to quantify the abundance of these bacterial taxa in all individual soil samples using the same qPCR chemistry and instrumentation as described above. Calibration curves were generated with serially diluted amplicons of a PCR reaction using the respective primers. In addition, abundance of ammonia oxidizers and the *AmoA* gene were measured in all soil samples using previously described primers (Kowalchuk et al., 1997; Rotthauwe et al., 1997) employing EvaGreen chemistry (5× HOT FIREPol EvaGreen qPCR Mix Plus-ROX), amplicon calibration, and the StepOnePlus Real-Time PCR System as described above with the reaction settings detailed in Supplementary Table S1.

### Calculation and Statistics

Plant P and N contents were calculated from biomass and nutrient concentrations data. Plant biomass, nutrient (N and P) concentrations, and nutrient content data as well as the root colonization by the AM fungi assessed microscopically or with qPCR were subjected to two-way analysis of variance (ANOVA) testing the influence of the AM fungal identity and harvest time. All data on the development of AM fungal hyphal networks in the different system compartments for each of the experimental units (pots) were standardized through division with the value of the hyphal development in the respective plant compartment (be it the hyphal length density or the concentration of DNA of a particular fungus in each of the pots). These values were further log (x+1) transformed to reduce heteroscedasticity in the data, thereby resulting in a variable termed “developmental response of AM hyphae to soil amendment,” and this was further subjected to ANOVA and correlation analysis using Statgraphics Plus 3.1 (Statpoint Technologies, Warrenton, VA, USA). The 454-sequencing data were analyzed separately for the eubacterial and fungal communities. The frequencies of reads belonging to particular clusters (OTUs) were normalized (i.e., expressed as a fraction of the total read number per sample), log-transformed, and subjected to principal coordinate analysis using Canoco for Windows v. 4.5 (Ter Braak and Šmilauer, 2002), based on the previously generated Bray-Curtis distance matrix. Microbial taxa showing significant positive or negative correlation of their relative abundance (as per 454-sequencing data) with the *R. irregularis* developmental response to soil amendments (one mean value per soil amendment used to achieve consistency with 454-sequencing data) were identified by a weighted multiple regression, using the *t*-value biplot approach in Canoco 5.04 (Šmilauer and Lepš, 2014) while adjusting the critical value of the test to |*t*| = 0.8458 due to the low number of samples (*n* = 8). The qPCR results from quantification of the bacterial taxa (and the *AmoA* gene) were expressed as per mg soil weight, log (x+1) transformed, then correlated (using a linear regression model) with the developmental response of the AM fungi as calculated above using Statgraphics Plus 3.1.

## Results

### Plant Growth and Nutrition, Root Colonization by AM Fungi

The plants grew slowly until the sixth week after sowing and then the growth rate increased markedly, producing several fold more biomass 9 weeks after sowing as compared to the harvest 3 weeks earlier (Supplementary Figure S2, Supplementary Table S2). Some of the early contrasts between the two AM fungal treatments, such as those observed in root P concentration and shoot N concentration 6 weeks after sowing (Supplementary Figure S3), vanished at the later harvest, resulting in a significant interaction term in the two-way ANOVA (Supplementary Table S2). However, other contrasts between the two AM fungi, such as that observed in root N concentration (Supplementary Figure S3, Supplementary Table S2), remained stable throughout the experiment.

The extent of root colonization by the two AM fungi was affected only by the identity of the fungus but not by the time of harvest (Supplementary Table S3). Means (for both harvests) of the fraction of root length colonized by hyphae, arbuscules, and vesicles for the *Rhizophagus*-inoculated plants were 74, 63, and 16%, respectively, and for *Claroideoglomus*-inoculated plants the means reached 57, 44, and 4%, respectively. Higher fungal colonization intensity of roots in the *Rhizophagus* as compared to the *Claroideoglomus* treatment was also confirmed by an independent qPCR approach (Supplementary Table S3).

### Development of AM Fungal Networks in the Soil

The pattern of AM fungal hyphae development in the soil, as assessed by microscopy, was affected by all three main factors, which is to say AM fungal identity, time of harvest, and identity of the soil patch. The pattern was affected (though less importantly) also by the interaction of AM fungal identity and soil patch as well as by the interaction of harvest time and soil patch (**Table 2**). Hyphal length densities in the plant compartment recorded microscopically were 0.57 ± 0.32 (standard deviation) m g^-1^, with individual pots showing values between 0.28 and 1.48 m g^-1^. No significant differences were detected in the absolute hyphal length densities in the plant compartment due to either AM fungal identity or harvest time or the interaction of these two factors.

**Table 2:** Results of three-way analysis of variance testing the effects of arbuscular mycorrhizal (AM) fungal identity, harvest time, and soil patch amendment on the development of AM fungal hyphae as assessed microscopically.

	*F*-value	*p*-value
AM fungal identity (A)	87.3	<0.001
Harvest time (B)	38.2	<0.001
Patch amendment (C)	6.29	<0.001
Interaction A × B	2.08	0.15
Interaction A × C	2.70	0.007
Interaction B × C	2.13	0.03
Interaction A × B × C	0.68	0.73

*Relative values with respect to plant compartment were analyzed, and these were further log (x+1) transformed for statistical comparisons. Four replicates were included in the analysis for each combination of factors.*

**FIGURE 1:**
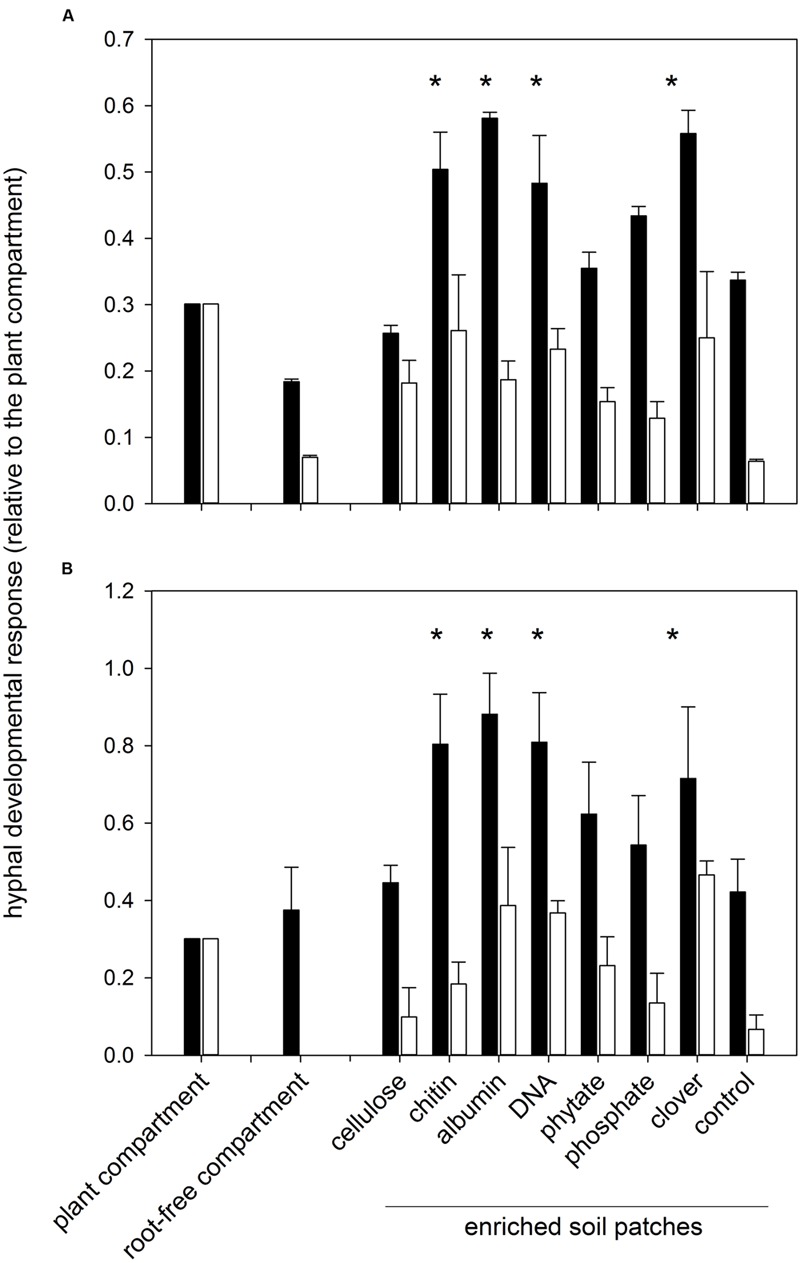
**Development of arbuscular mycorrhizal (AM) fungal hyphae in different system compartments as assessed microscopically (**A**) and using quantitative real-time PCR (qPCR; **B**).** Shown are relative values with respect to plant compartment which were further log (x+1) transformed for statistical comparisons. Means +1 standard error are shown. Combined microscopic data across both harvests (6 and 9 weeks) are shown in **(A)**, whereas the molecular data **(B)** were collected at the second harvest. Black bars represent pots inoculated with *Rhizophagus irregularis* and white bars pots with *Claroideoglomus claroideum*. Treatments marked with an asterisk indicate a consistent positive effect of the soil amendment on mycorrhizal hyphal development (*p* < 0.05) as compared to the control (non-amended) soil patch, according to three- or two-way analysis of variance [for **(A,B)**, respectively] and mean separation with Fisher’s *F*-test.

Greater root-free compartment colonization by AM fungal hyphae relative to the development of the hyphae in the plant compartment was observed in the *Rhizophagus*-inoculated pots as compared to the *Claroideoglomus* pots (**Figure 1**), driving the effect of AM fungal identity (**Table 2**). Generally greater hyphal abundance was also observed in the second harvest as compared to the first, thereby indicating gradual colonization of the root-free compartment as compared to the plant compartment, with the latter being used as a denominator in the hyphal response variable (**Table 2**). Using three-way ANOVA, the effect of soil patch identity was consistent across both AM fungal inoculation treatments and the times of harvest, with chitin, albumin, DNA, and clover biomass significantly stimulating the development of AM fungal networks as compared to the control (non-amended) soil patch (**Table 2** and **Figure 1**). Because there were two significant two-way interactions involving the soil patch effect (**Table 2**), these required a more detailed analysis.

First, whereas there were certain soil patches (i.e., those amended with chitin, albumin, DNA, and clover biomass) where the recorded AM hyphal density significantly exceeded the values in the plant compartment for the *Rhizophagus* pots, the hyphal densities in the soil patches of the *Claroideoglomus* pots never significantly exceeded the values recorded in the plant compartment. These differences in colonization of the root and root-free compartments drove the significance of the interaction term between AM fungal identity and the identity of soil patches in the three-way ANOVA comparison (**Table 2**). Second, whereas there was a significant effect of the soil patch quality on the AM hyphal development recorded across both harvests (**Table 2** and **Figure 1**), mainly driven by the values recorded in the second harvest (*F*_9,79_ = 4.17, *p* < 0.001), no significant differences in AM hyphal response to the different soil patches were recorded at the first harvest. This dynamic explains the significance of the interaction term between the soil patch identity and the time of harvest (**Table 2**).

**FIGURE 2:**
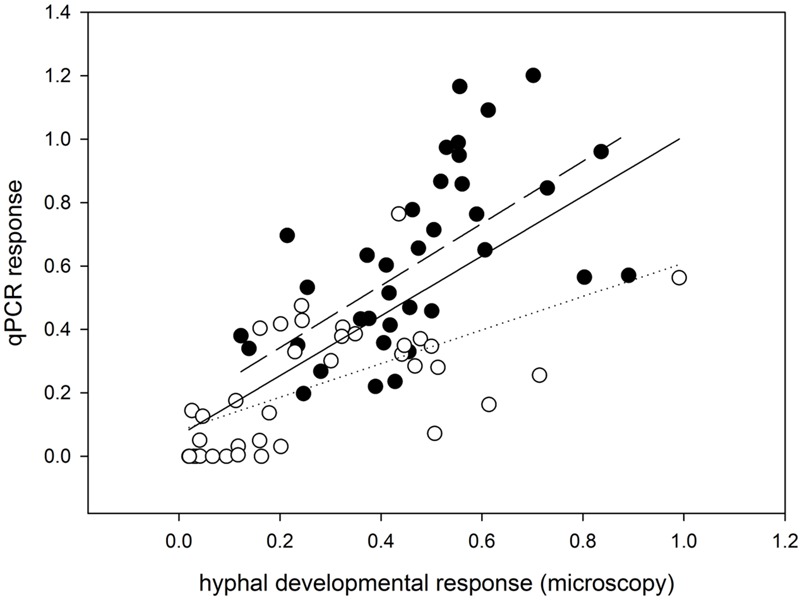
**Correlation analysis showing consistency of the developmental response of mycorrhizal fungal hyphae in the different pot compartments (including the rooted and root-free compartments and the differentially enriched soil patches) as assessed microscopically (x-axis) and by qPCR (y-axis).** Data were standardized with the values obtained from respective plant compartments and further log (x+1) transformed for the statistical analysis. Data and regression line for *R. irregularis*-inoculated pots are shown as closed circles and a dashed line; data and regression for pots with *C. claroideum* are shown as open circles and a dotted line (*R^2^* = 0.47 for *R. irregularis* and *R^2^* = 0.39 for *C. claroideum*). Regression line from the analysis pooling all data together is shown as a solid line (*R^2^* = 0.37). All linear regressions are highly significant, with *p* < 0.001.

Importantly, the effects recorded by microscopy were largely confirmed using qPCR with AM-taxa specific markers on the soil DNA extracts from the pots harvested 9 weeks after sowing (compare **Figure 1A** and **Figure 1B** and see also Supplementary Figure S4). In addition, there were significant correlations between the AM hyphal responses as recorded microscopically and by molecular quantification (**Figure 2**) for each of the AM fungal taxa separately and even when pooled.

### Microbial Communities in the Soil Patches

**FIGURE 3:**
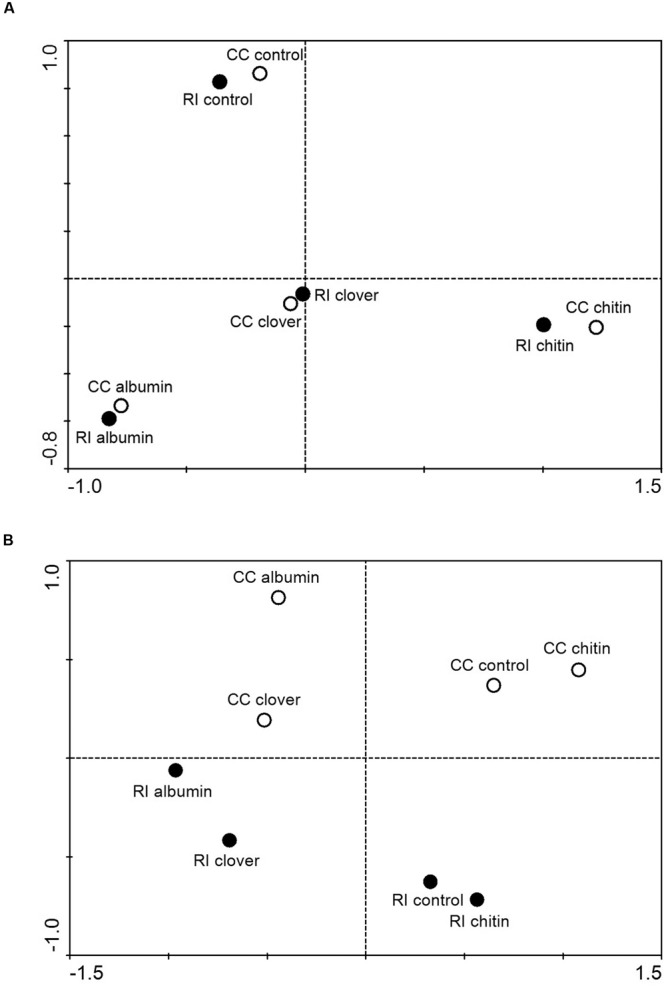
**Results of principal coordinate analysis depicting similarities between prokaryotic **(A)** and fungal **(B)** communities in soil patches amended or not amended with various organic compounds and colonized by *R. irregularis* (RI) or *C. claroideum* (CC).** DNA extracts from replicated patches of the same kind were pooled, amplified with group-specific primers, labeled with multiplex identifier adaptors, and subjected to 454-amplicon sequencing. Sequences were trimmed to 400 bp length, denoised, cleared of chimeras, and clustered at 95% (prokaryotes) or 97% (fungi) similarity levels. Singletons were removed and the relative abundances of operational taxonomical units (OTUs) calculated, fungal OTUs with mean abundance across the different samples below 0.25% and prokaryotic OTUs with abundance below 0.1% removed from the dataset, the abundances log-transformed, and Bray-Curtis distance matrices calculated. Through the filtering procedure (due to their low abundance), all Glomeromycota sequences present in the fungal dataset were removed prior to analysis.

The 454-sequencing returned 80,013 prokaryotic and 88,722 fungal sequence reads (after quality filtering), which clustered to 3,323 bacterial OTUs and 704 fungal OTUs. The numbers of OTUs entering the statistical analyses (without molecular singletons) were 1,591 for bacteria and 361 for fungi (Supplementary Table S4). Principal coordinate analysis revealed a strong effect of the quality of soil patch amendments on the bacterial communities and a nearly negligible effect of AM fungal identity (**Figure 3A**), whereas both the quality of soil amendment and the identity of the AM fungus obviously played roles in the structuring of the fungal communities (**Figure 3B**). It is important to note here that the sequenced fungal communities contained only a very small fraction of sequences assigned to AM fungi (<4.5%) and of this fraction the vast majority (>98%) were identified as *R. irregularis* (or *Glomus intraradices*) and only a few dozen sequences were identified as *Claroideoglomus* (or earlier synonyms). Abundances of dominant bacterial and fungal taxa in the 454-sequencing profiles of the different samples are shown in the Supplementary Material (Supplementary Figures S5 and S6).

*T*-value biplot analysis identified 13 bacterial OTUs that significantly (*p* < 0.05) and positively correlated with *Rhizophagus* hyphal response in the soil patches as assessed by qPCR, 28 bacterial OTUs that correlated negatively, as well as 1 fungal OTU correlating positively (*Melampsora epitea*) and 1 fungal OTU (*Penicillium raistrickii*) correlating negatively (Supplementary Table S5). Among the positively correlating bacterial taxa, four of the strongly correlating OTUs were identified as *Nitrosospira* sp., and two (of the less strongly yet significantly correlating) OTUs as a bacterium similar to *Acanthamoeba* endosymbiont (Supplementary Table S5). Further analyses of the supposed *Acanthamoeba* endosymbiont sequences showed that the two OTUs represented forward and reverse reads of one amplicon (624 bp long) with a very high sequence similarity (98–99% across the full length except the forward priming site) to an obligate protist endosymbiont Candidatus *Caedibacter acanthamoebae*.

**FIGURE 4:**
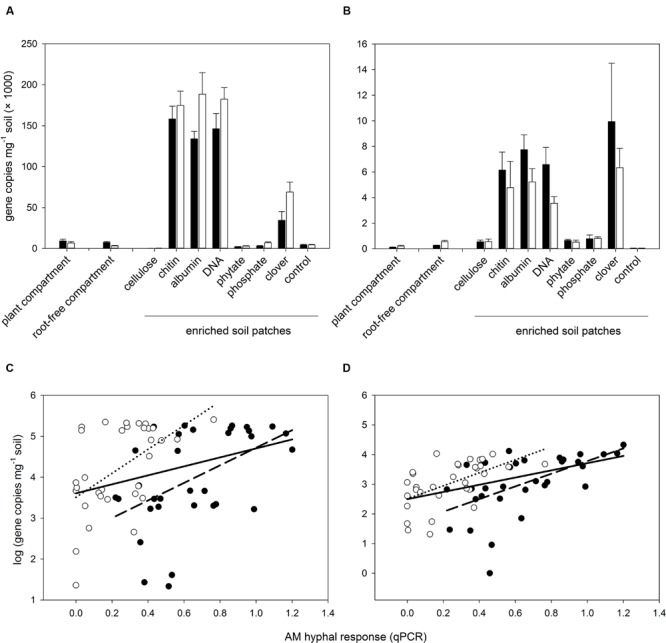
**Abundance of selected prokaryotic taxa (*Nitrosospira* sp. – **A**, and a prokaryote similar to *Acanthamoeba* endosymbiont – **C**) in the soil of different system compartments as detected by novel qPCR markers (see Supplementary Table S1 for details) and correlation between abundance of the two prokaryotic taxa and the developmental response of AM fungal hyphae to the different soil amendments in the root-free patches (**B,D**, respectively) assessed by qPCR with AM taxa-specific markers.** Black bars, closed circles, and dashed regression lines refer to pots inoculated with *R. irregularis*, whereas white bars, open symbols, and dotted regression lines refer to pots inoculated with *C. claroideum*. Bars represent means (*n* = 4) with associated standard errors. Solid regression lines show the correlations of all data pooled across the two inoculation treatments. All plotted correlations were significant at the level of *p* < 0.05 or stronger.

Subsequent qPCR analyses of all individual soil samples with specific markers for the above two prokaryotic taxa (*Nitrosospira* sp. and *Acanthamoeba* endosymbiont) revealed their much increased abundance in organic-N enriched soil patches as well as significant correlations of their abundance with the AM hyphal response to the soil amendments (**Figure 4**). Very similar patterns of abundance (with somewhat higher values in *Claroideoglomus*-inoculated pots as compared with *Rhizophagus*-inoculated pots) were also observed for the ammonia oxidizer populations analyzed by qPCR using previously published primers as well as for the abundance of the *AmoA* gene in the soil (Supplementary Figure S7).

## Discussion

Here we show, using a multiple-choice experiment, specific and consistent stimulation of AM hyphal development in soil patches amended with N-containing organic compounds, whereas other amendments (particularly the phytate and orthophosphate, but also cellulose) caused no localized hyphal response in the same fungal colony. These results lead to rejection of the original hypothesis predicting preferential colonization of P-containing patches due to high P demand of the N-unlimited host. Although the observed absence of AM hyphal growth stimulation by cellulose just confirms previous observations (Ravnskov et al., 1999; Gryndler et al., 2002), no obvious effect of P amendments on the AM hyphae is surprising. It is namely inconsistent with previous studies demonstrating stimulation of AM hyphal growth by phytate and by low levels of inorganic orthophosphate (Feng et al., 2004; Cavagnaro et al., 2005; Zheng et al., 2015). Thus, our results deserve detailed analysis and possibly further experimentation to find definitive answers about the nature of AM hyphal growth stimulation and mineral nutrient acquisition from root-free patches of different qualities.

One possible explanation for the lack of AM hyphal growth stimulation by the P-containing amendments could be that the AM fungal P uptake efficiency from the soil solution is partly or totally uncoupled from the hyphal development. However, based on previous research (e.g., Jansa et al., 2005), this is highly unlikely, although we admit that a direct radiophosphorus labeling of the P amendments and including non-mycorrhizal controls would be required to provide a definitive answer to this issue.

The most intriguing question is thus why both the AM fungi so clearly and consistently proliferated in the organic N patches in our experiment. Although, half of the stimulatory soil amendments (i.e., DNA and clover biomass) also contained P (**Table 1**), P alone is unlikely to have caused the observed AM hyphal stimulation. This is because the amounts of DNA or clover biomass used in creating the stimulatory patches contained the same or smaller amounts of added P than did the phytate or inorganic orthophosphate patches, which in turn caused no significant stimulation of AM hyphal development in soil (see above). Given our experimental setup and the (N-fixing) model plant, the requirements of the host plant for soil-derived N should be rather low (Sulieman et al., 2013) compared to previous studies with non-leguminous hosts such as *Plantago* (e.g., Hodge et al., 2001; Gryndler et al., 2003). On the other hand, some earlier studies (e.g., Ravnskov et al., 1999) using clover (another leguminous and N-fixing plant) as a host also demonstrated stimulation of the AM hyphal networks by localized organic N amendments. Together, these results strongly suggest that it is primarily the AM fungal requirement for N rather than plant N demand, which drives the observed AM hyphal proliferation in organic N patches. This notion is not inconsistent with the previous research clearly demonstrating transfer of the N (but never the C) from the organic matter via AM hyphae to the associated mycorrhizal (and N-limited) host plant (Hodge et al., 2000, 2001; Hodge and Fitter, 2010; Herman et al., 2012). However, our results call for a more myco-centric view (e.g., Alberton et al., 2005) on the AM symbiosis, where the requirements of both partners and possible competition between the partners for limited resources such as mineral N is considered (Hodge and Storer, 2015). One particularly interesting issue in this regard is whether N-unlimited host would transfer any N to its associated (and N-limited) AM fungus – to the best of our knowledge there is no experimental evidence for this as yet.

Although, we did use neither direct ^15^N labeling of the organic amendments nor we included non-mycorrhizal controls into our experiment, our ^15^N abundance results indicate that the N taken up from soil by *Rhizophagus* resides mainly in the fungal biomass and is not transferred to the plant host. This is because the ^15^N signature of the *Rhizophagus-*colonized roots was (across both harvests) higher than that of the roots colonized by *Claroideoglomus (p* = 0.043, see Supplementary Figure S8 for data). On the other hand, the ^15^N signature of the shoots of *Rhizophagus*-colonized plants was much lower than that of the *Claroideoglomus-*inoculated plants (*p* < 0.001, Supplementary Figure S8). The latter was most likely due to markedly improved P nutrition of the *Rhizophagus*-colonized plants having positive feedback on the highly P-dependent symbiotic N fixation (Scheublin et al., 2004). Because positive values usually indicate N taken from the soil and/or fertilizer pool, lower values indicate N derived from biological N fixation (George et al., 1993). *Rhizophagus* colonized the roots more heavily than did *Claroideoglomus* in our experiment, and it also provided greater growth and nutritional benefits to its hosts. Thus, our results indirectly indicate greater biological N fixation of the *Rhizophagus*-inoculated plants, whereas most of the N taken up by the fungus is likely not transferred to the plant tissues.

Because the saprotrophic potential of the AM fungi is thought to be low and cannot alone explain the degradation of soil organic amendments (Joner et al., 2000; Koide and Kabir, 2000; Leigh et al., 2011), the AM hyphae most likely benefit from/depend on the degradatory activities of such other soil microbes as bacteria (Beier and Bertilsson, 2013) to access nutrients such as N in organic forms. Once in the soil solution, the mineral N ions can be directly taken up by AM fungal hyphae (Bago et al., 1996; Govindarajulu et al., 2005; Cruz et al., 2007). There are always plenty of microbes both in the soil and on the surfaces of AM hyphae, and their communities can also actively be shaped by AM fungal hyphae (Gryndler et al., 2003; Toljander et al., 2006, 2007; Drigo et al., 2010; Jansa et al., 2013; Nuccio et al., 2013). Thus, the possible feedbacks between proliferation of AM hyphae, soil mineral N forms, and the soil microflora in the enriched soil patches need particular attention here.

Previously, it has been shown that addition of buffered ammonia could have triggered hyphal branching of *R. irregularis* in absence of any bacteria in a monoxenic cultivation system (Bago et al., 2004). Open pot experiments (e.g., Gavito and Olsson, 2008), could not replicate the same phenomenon, however, and so it remains unclear whether the previous results could be generalized for *Claroideoglomus* due to substantial functional differences in the hyphal growth traits between the two AM fungal genera (Thonar et al., 2011). Inasmuch as the stimulatory patches in our experiment were all enriched with organic N sources (with the exception of clover biomass, where a small portion of N could have been present as nitrate or ammonia already from the very beginning), release of mineral N forms would have required exoenzyme activity, thus implicating soil saprotrophs as being involved in the AM hyphal response to soil patches. This would be consistent with previous findings showing substantial stimulation of AM hyphal proliferation by some soil bacteria (Gryndler et al., 2000), although other studies have also reported antagonistic interactions between AM fungi and soil bacteria (e.g., Leigh et al., 2011). In our study, the dominant OTUs of the 454-sequencing profiles of both bacterial and fungal communities in the different soil patches did not consistently explain the AM hyphal responses to the patches but rather mirrored the different patch qualities in an idiosyncratic manner (see Supplementary Figures S5 and S6). On the other hand, several bacterial taxa with lower abundance in the 454-sequencing profiles showed significant positive correlation with the *Rhizophagus* hyphal responses to the soil amendments, and this was also confirmed by the qPCR analyses including all individual soil samples (**Figure 4**). First, strong correlation of the hyphal response with some specific *Nitrosospira* sp. abundance across the different soil amendment types indicates the possibility of a causal (nutritional or signaling) relationship. This is because this bacterial genus has the capacity to oxidize ions of ammonium to nitrite in the first step of nitrification and often dominates this ecosystem function (Webster et al., 2005). It may be important to note that OTUs representing other microbes involved in nitrification (e.g., *Nitrobacter, Nitrospira, Nitrosovibrio*, as well as other *Nitrosospira* spp.-like OTUs; Supplementary Table S4) did not correlate with AM hyphal response. Second, the correlation with the *Acanthamoeba* endosymbiont may indicate an involvement of protozoa in the stimulation of AM hyphal growth within the soil patches. Protozoa mobilize N from bacterial biomass and have recently been shown to increase the rates of N translocation from organic fertilizers to plants via the mycorrhizal pathway (Koller et al., 2013). However, the correlation, no matter how strong, does not constitute causal proof, which would need to be obtained by means of specifically focused experiments yet to come. These future experiments should also enable testing as to whether the interactions of AM fungi with protozoa and/or *Nitrosospira* and possibly other prokaryotes involved in nitrification indeed center upon the N nutrition of the AM fungus or the plant, or whether it could also involve secondary metabolites or one or more of the other degradation products resulting from the enzymatic hydrolysis of the organic amendments.

Relatively low AM hyphal densities in the soil samples collected from the different compartments in this study as compared to other, similar studies (e.g., Thonar et al., 2011) could possibly be explained by the fact that we used granular zeolite (expanded clay) as a substantial part (45% volume) of our potting substrate. This material is porous and there is evidence that the AM fungi would spread their hyphae inside the pores and cavities of the granules (Baltruschat, 1987; Feldmann and Idczak, 1992). Any hyphae inside the granules would not be accessible to mechanical hyphae extraction and microscopy. However, inasmuch as our DNA-based quantification correlates well with the patterns of AM hyphal spread in the different compartments observed microscopically (**Figure 2**) and the DNA should extract all fungal tissues, both inside and outside the zeolite granules, we are confident as to the validity of the hyphal developmental responses reported in this study.

## Conclusion

We found a very strong evidence of AM hyphal proliferation in organic N but not in organic or inorganic P patches, in spite of the fact that our host plant (*M. truncatula*) was not N limited (as inferred from similar experiments using the same soil, AM fungi and plant genotype as here, e.g., Konvalinková et al., 2015). Thus, AM hyphal proliferation seems to be caused primarily by the N requirements of the AM fungus rather than the host plant. We consider our results to be particularly robust because we used two independent approaches to quantify the development of AM fungi in soil, namely the traditional microscopy and the qPCR. To our knowledge, this is the first study showing a fair correlation between these two independent methods quantifying the development of AM fungal hyphae in soil. This correlation is particularly important inasmuch as there was a significant stimulation of other soil fungi’s development in certain soil patches and microscopic quantification of the hyphal development could potentially be biased by our inability to discriminate between AM fungal and other hyphae. Using two independent approaches (454-sequencing of pooled soil samples per soil amendment treatment and qPCR using all individual soil samples), we also demonstrated that the hyphal developmental responses of both AM fungi to soil amendments strongly correlated with the abundance of *Nitrosospira* sp., an ammonium oxidizer, and few other bacterial taxa in the soil including an obligate *Acanthamoeba* endosymbiont. This last evidence, though only correlative, strongly suggests a possible involvement of soil protists such as *Acanthamoeba* sp. in mediating the stimulation of AM hyphal development by and possibly hyphal uptake of N from the soil organic-N amendments, possibly through so called microbial loop (Bonkowski, 2004). Both the amoebas and the ammonia oxidizers could be part of such a pathway – with the ammonia oxidizers processing either the NH_4_^+^ ions released directly from the organic matter (scenario 1) or the ammonia accumulating in the soil solution as a by-product of amoebas grazing on soil bacteria (scenario 2). The latter appears much more plausible scenario than the previously suggested primary involvement of ammonia oxidizers in oxidation of ammonia directly released from the organic matter (Cheng et al., 2012). This earlier work established the scenario 1 based on the observed repression of AM-induced organic matter mineralization by nitrification inhibitor dicyandiamine – silently assuming that the AM fungi would be generally very inefficient in taking up the free NH_4_^+^ ions from the soil solution. Such an assertion is, however, not supported by most previous research (e.g., Govindarajulu et al., 2005). Either way, complex microbial interactions involved in organic matter mineralization and AM fungal uptake of N released by this process certainly warrant further investigations, addressing possible metabolic/trophic dependencies between the different members of soil microbial communities, as well as consequences of these interactions for the N nutrition of the AM fungus and its host plant. More dedicated efforts with ^15^N-labeled sources (be they atmospheric N_2_, soil organic soil amendments, or mineral N fertilizers), specific non-mycorrhizal and non-microbial controls and intensive sampling schemes covering temporal dynamics of microbial communities will be required to quantify precisely the contributions of the various microbial pathways to and involvement of the different players in the plant and AM fungal nutrition.

## Author Contributions

PB, MG, and JJ conceived the research, PB, JJ, and DP planned the experiment, PB, DP, and HG conducted the pot experiment and most of the associated analyses, PB and MG conducted the 454-sequencing including the bioinformatics analyses, JJ designed the new qPCR markers, PB conducted and evaluated the qPCR analyses, all authors contributed to writing and agreed with the final form of the manuscript before submission.

## Conflict of Interest Statement

The authors declare that the research was conducted in the absence of any commercial or financial relationships that could be construed as a potential conflict of interest.

## Footnotes

1 http://www.zeopol.com

2 http://trace.ncbi.nlm.nih.gov/Traces/sra/
