# Supplementary material for: Organic Nitrogen-Driven Stimulation of Arbuscular Mycorrhizal Fungal Hyphae Correlates with Abundance of Ammonia Oxidizers
Source: Front Microbiol. 2016 May 12;7:711. doi: 10.3389/fmicb.2016.00711 (PMC4863899; doi:10.3389/fmicb.2016.00711)

Supplementary Material

Organic nitrogen-driven stimulation of arbuscular mycorrhizal fungal hyphae correlates with abundance of ammonia oxidizers

Authors: Petra Bukovská, Milan Gryndler, Hana Gryndlerová, David Püschel, Jan Jansa*

Affiliation: Laboratory of Fungal Biology, Institute of Microbiology, Czech Academy of Sciences, Prague, Czech Republic

***Correspondence:**

Jan Jansa

jansa@biomed.cas.cz

Table S1| Quantitative real-time PCR markers (newly designed AOB2 and AAE targeting ammonium oxidizing bacterium closely related to *Nitrosospira* and *Acanthamoeba* endosymbiont, respectively) and previously described degenerated CTO primers used for quantification of selected bacterial taxa and ammonium monooygenase structural gene AmoA (*AmoA*) in soil samples. PCR cycling followed initial denaturation at 95°C for 12–15 min, 50–60 cycles with denaturation at 95°C for 10–15 s, annealing as specified below, and elongation at 72°C for 20–60 s each cycle. Following amplification, EvaGreen-based detection methods (i.e., quantification of CTO and *AmoA* gene) were followed by melting curve analyses consisting of denaturation at 95°C for 15 s, re-association at 60°C for 60 s, temperature gradient at 0.3°C/s, and final denaturation at 95°C for 15 s.

Marker	Target	Sequences (5'–3'):<br>forward primer,<br>reverse primers,<br>TaqMan probe (if applicable)	Amplicon length (bp)	Annealing temperature (°C)	Annealing time (s)	Reference
AOB2	*Nitrosospira* sp.	GCAGGCGGTTTTGTAAGTC<br>CCACATCTCTACGCATTTCAC<br>CCTCTGCCACACTCTAGCCTTG	131	53	40	This study
AAE	bacterium similar to *Acanthamoeba* endosymbiont	TGGATGACTAGAGGACGAGAG<br>CGCCTCAGTGTCAGATACG<br>AAAGCCGCCTTCGCCTCTG	120	52	45	This study
CTO	ammonia oxidizers	GGAGRAAAGYAGGGGATCG<br>CTAGCYTTGTAGTTTCAAACGC	465	57	40	Kowalchuk et al. 1997, Applied and Environmental Microbiology 63:1489-1497
AmoA gene	ammonia oxidizers	GGGGTTTCTACTGGTGGT<br>CCCCTCKGSAAAGCCTTCTTC	491	58	30	Rotthauwe et al. 1997, Applied and Environmental Microbiology 63:4704-4712

Table S2 | Results of two-way analysis of variance testing the effects of arbuscular mycorrhizal (AM) fungal identity and harvest time on the biomass production, above- to belowground biomass partitioning of the *Medicago truncatula* plants, and phosphorus (P) and nitrogen (N) concentrations and contents in the plants. Four replicate values were included in the analysis for each combination of factors. *F*-values are shown with indication of statistical significance, ns – $p \geq 0.05$, * – $0.05 > p \geq 0.01$, ** – $0.01 > p \geq 0.001$, *** – $p < 0.001$.

	AM fungal taxon (A)	Harvest time (B)	Interaction (A × B)
Plant dry biomass (shoot and roots combined)	86.3 ***	457.6 ***	53.5 ***
Biomass partitioning (root to shoot ratio)	0.23 ns	1.92 ns	0.0 ns
Plant P content (mg)	86.7 ***	126.7 ***	19.7 ***
Plant N content (mg)	54.1 ***	271.1 ***	29.3 ***
Shoot P concentration (mg g^{-1})	10.8 **	25.8 ***	3.46 ns
Root P concentration (mg g^{-1})	10.2 **	101.8 ***	11.8 **
Shoot N concentration (mg g^{-1})	2.46 ns	29.3 ***	5.6 *
Root N concentration (mg g^{-1})	19.8 ***	1.3 ns	1.7 ns

Table S3 | Results of two-way analysis of variance testing the effects of arbuscular mycorrhizal (AM) fungal identity and harvest time on the extent of root colonization by hyphae, arbuscules, and vesicles, as well as on quantification of the two AM fungi in the roots with taxon-specific quantitative real-time PCR (qPCR) markers. Four replicate values were included in the analysis for each combination of factors. *F*-values are shown with indication of statistical significance, ns – $p \geq 0.05$, * – $0.05 > p \geq 0.01$, ** – $p < 0.01$.

	AM fungal taxon (A)	Harvest time (B)	Interaction (A × B)
Fraction of root length colonized by AM fungal hyphae	7.30 *	0.2 ns	0.53 ns
Fraction of root length colonized by arbuscules	8.74 *	0.37 ns	0.28 ns
Fraction of root length colonized by vesicles	16.2 **	1.31 ns	2.20 ns
qPCR quantification (ng genomic DNA of a particular AM fungal taxon mg^{-1} dry roots)	18.2 **	0.93 ns	1.24 ns

Figure S1 | Arrangement of enriched patches enwrapped in 100 µm mesh around a central plant compartment (500 ml volume) fabricated from 40 µm mesh (**A**) and cultivation of *Medicago truncatula* plants in the greenhouse (**B**).

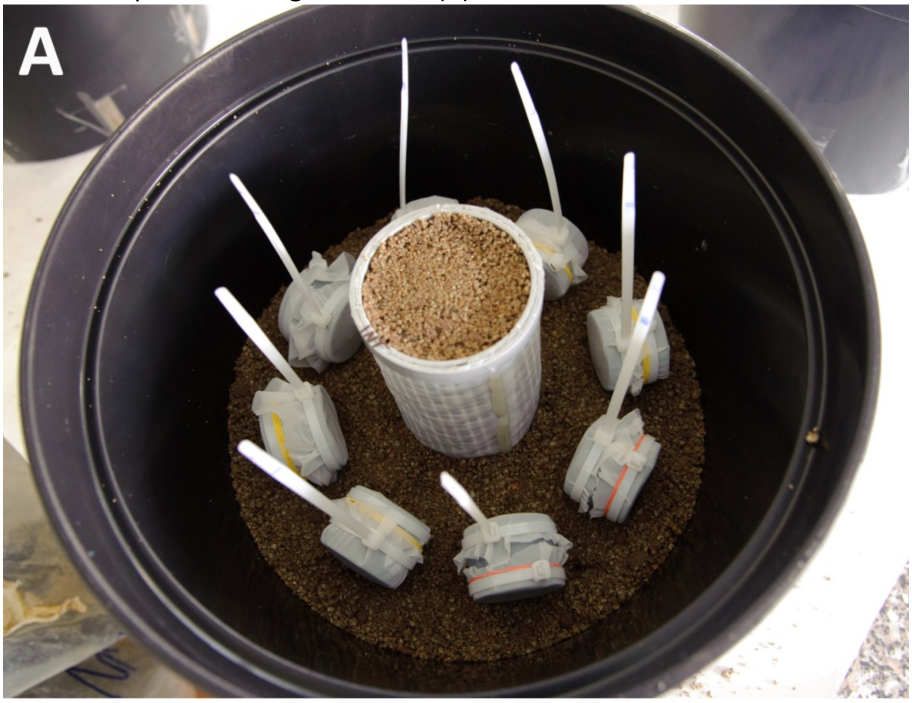

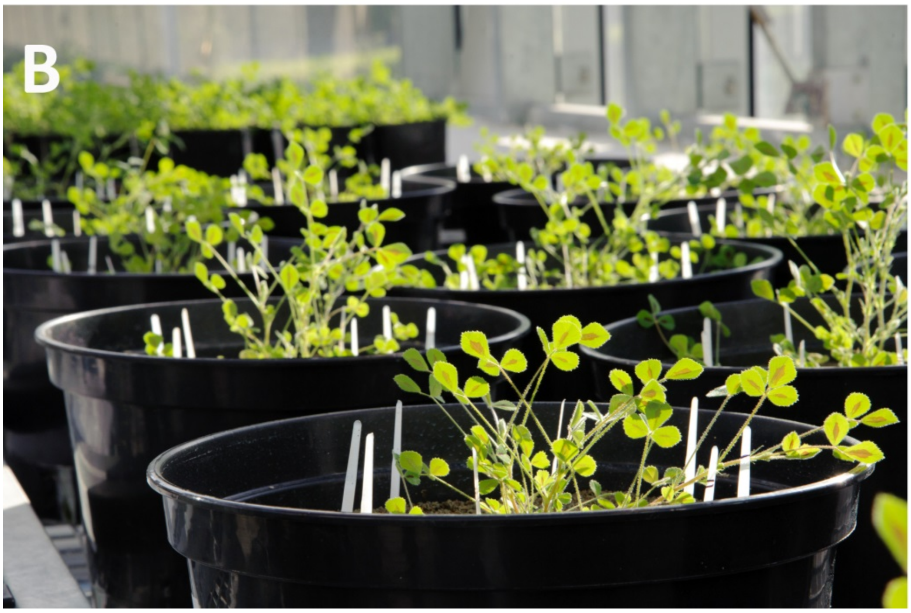

Figure S2 | Dry biomass (**A**), total phosphorus content (**B**), and total nitrogen content (**C**) of *Medicago truncatula* plants as affected by the identity of arbuscular mycorrhizal fungus and time of harvest. Values for shoots and roots were combined for presentation. Black bars – plants colonized by *Rhizophagus irregularis*, white bars – plants colonized by *Claroideoglomus claroideum*. Shown are means +1 standard error.

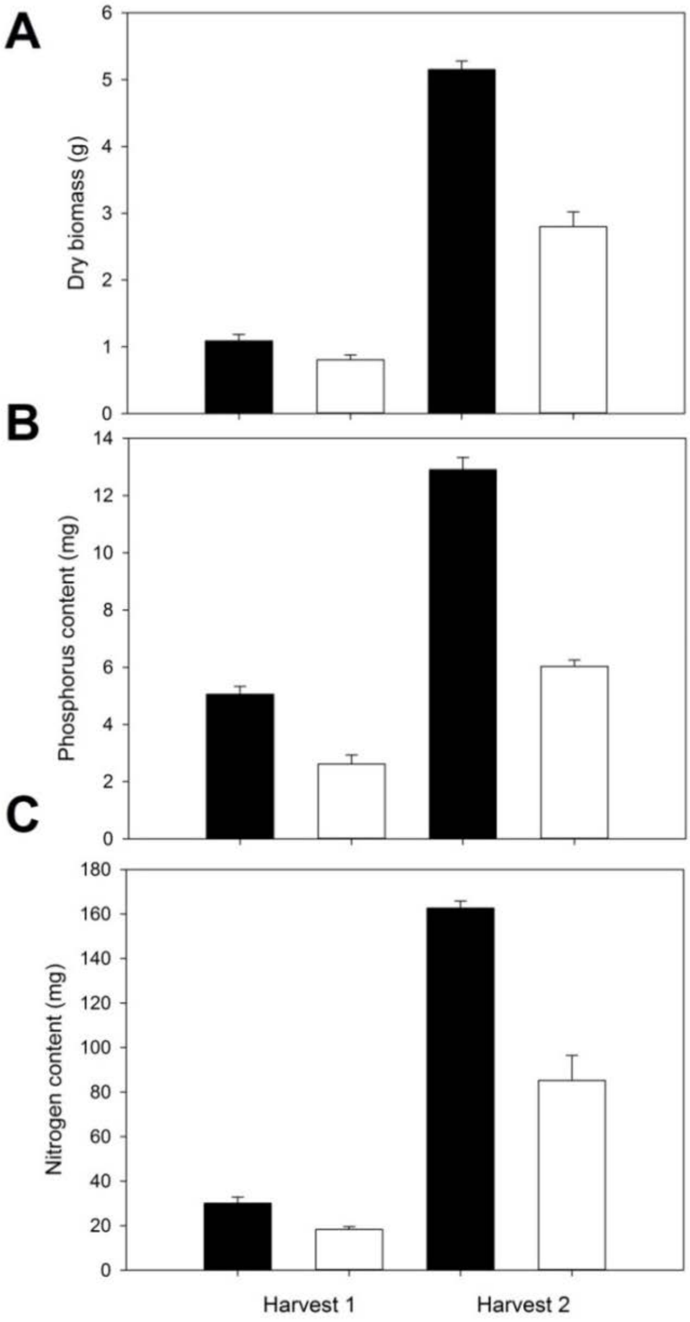

Figure S3 | Phosphorus (P; **A** and **B**) and nitrogen (N; **C** and **D**) concentrations in the shoots (**A** and **C**) and roots (**B** and **D**) of the *Medicago truncatula* plants as affected by the identity of arbuscular mycorrhizal fungus and time of harvest. Black bars – plants colonized by *Rhizophagus irregularis*, white bars – plants colonized by *Claroideoglomus claroideum*. Shown are means +1 standard error.

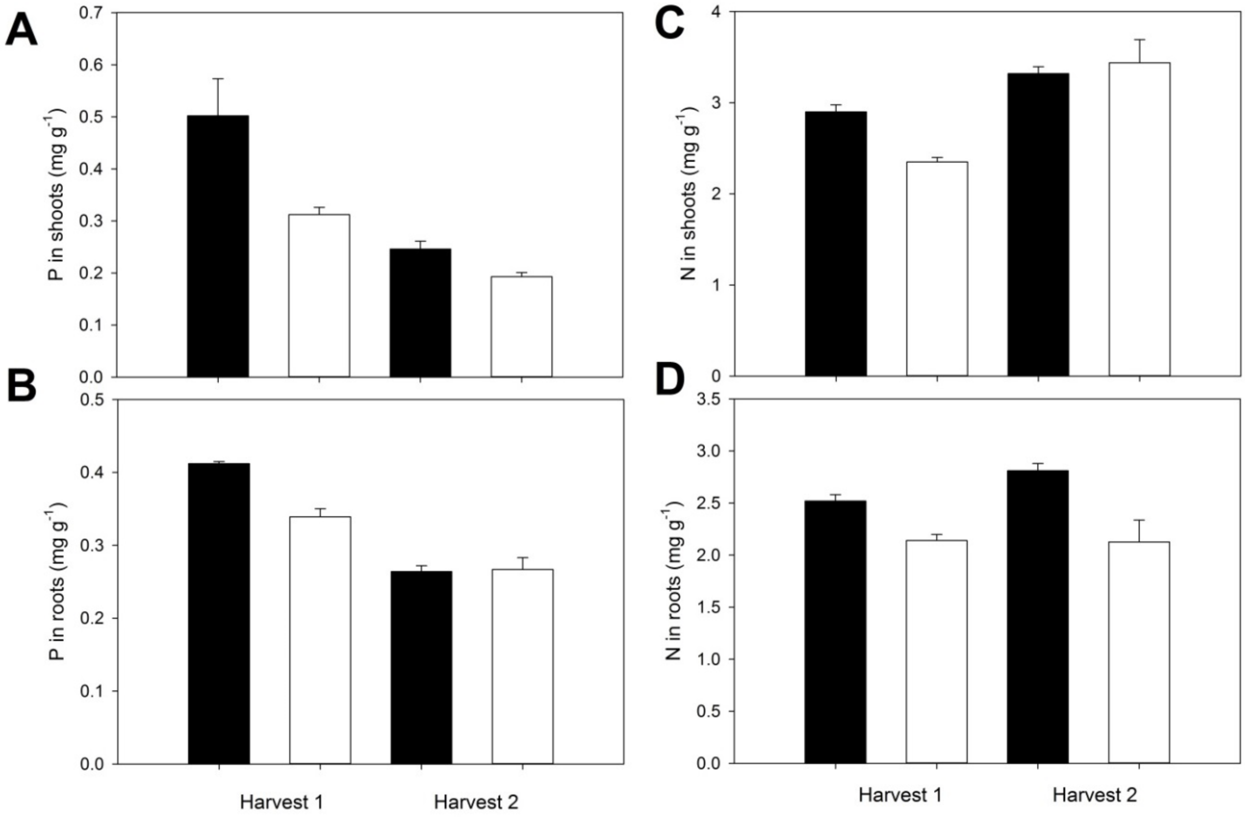

Figure S4 | Abundance of *Rhizophagus irregularis* (black bars) and *Claroideoglomus claroideum* (white bars) in the soil of different compartments of pots inoculated with *R. irregularis* and *C. claroideum* at the second harvest, as measured by quantitative real-time PCR. Nuclear large ribosomal subunit gene concentrations of the two arbuscular mycorrhizal fungal taxa were obtained using taxon-specific quantitative real-time PCR markers with TaqMan probes and corrected for DNA losses as assessed with internal DNA standard recovery analysis. Shown are treatment means and associated standard errors (*n* = 4).

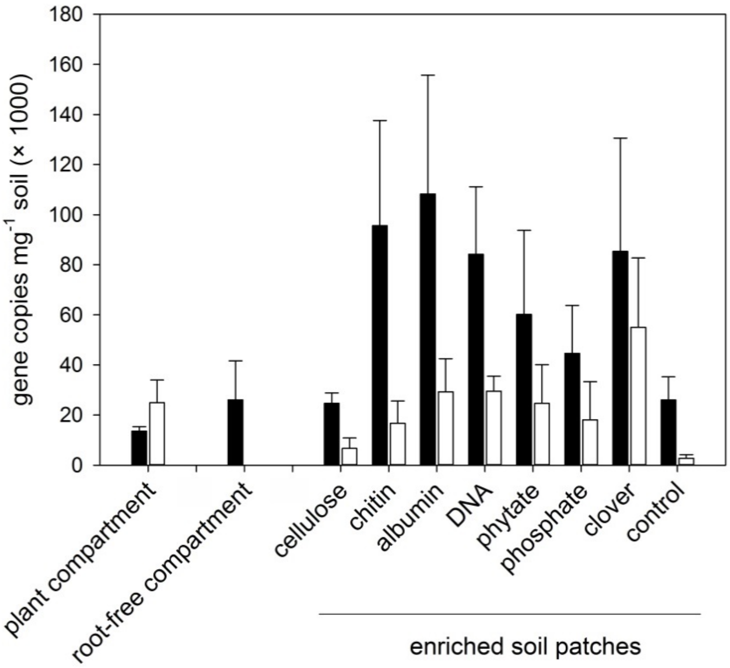

Figure S5 | Profiles of prokaryotic (A) and fungal (B) communities in the soil patches colonized either by *Rhizophagus irregularis* (RI) or *Claroideoglomus claroideum* (CC) and amended or not amended by three different organic compounds. Molecular singletons were removed from the operational taxonomic unit (OTU) list and relative abundances in each sample were calculated. Only those OTUs with relative abundance >5% in at least one sample are shown. The OTUs (cluster numbers shown in the legend) identified as the same genus were merged for presentation. Gray bars indicate the proportion of identified OTUs which did not fulfil the criteria for inclusion into the figure.

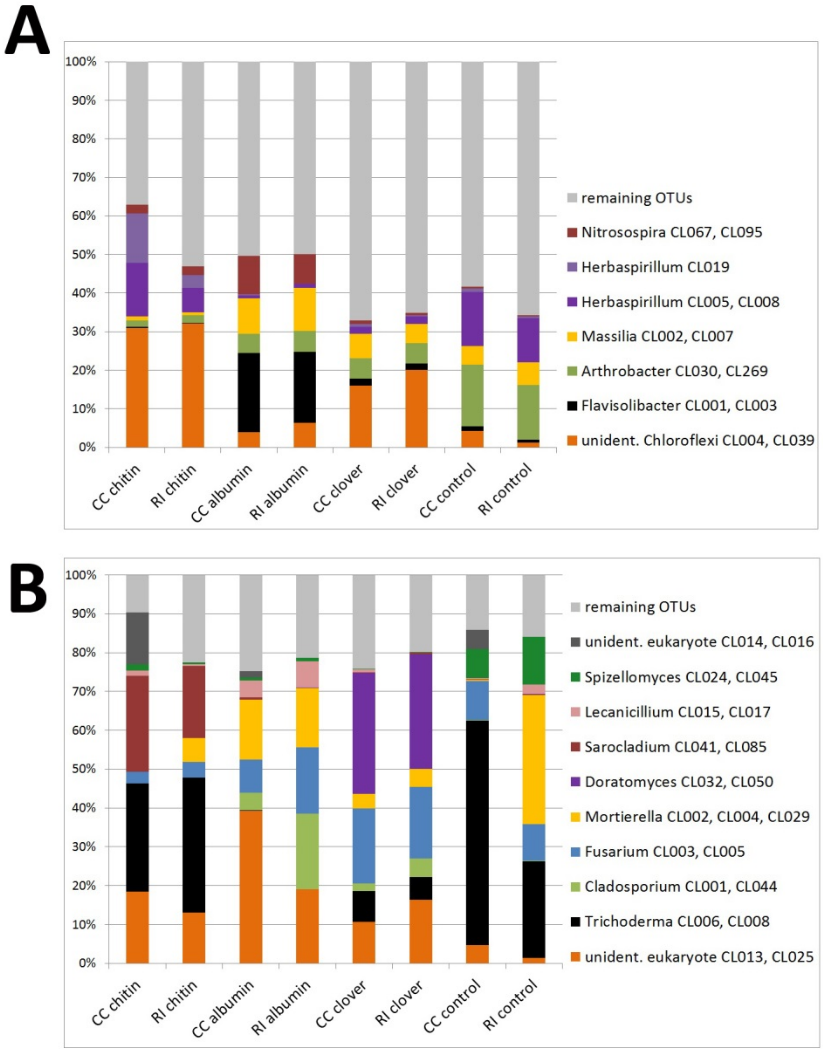

Figure S6 | Profiles of prokaryotic (**A**) and fungal (**B**) communities in soil patches colonized by *Rhizophagus irregularis* and supplemented or not supplemented with a wide range of soil amendments. For further details, see the legend to Supplementary Figure S5.

A

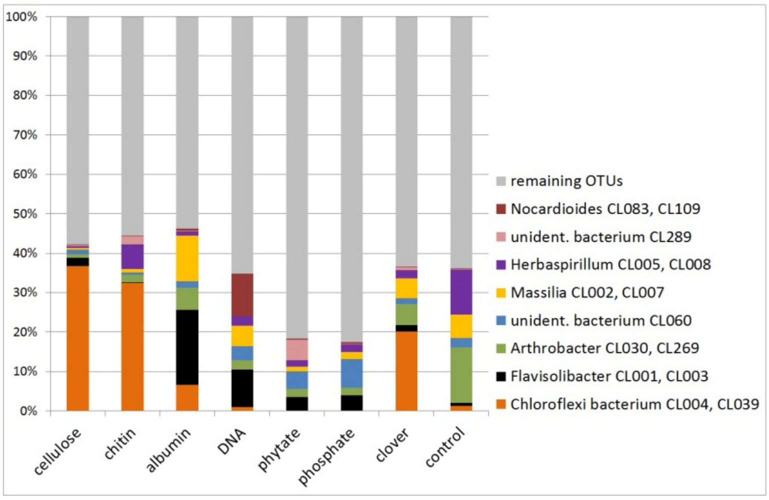

B

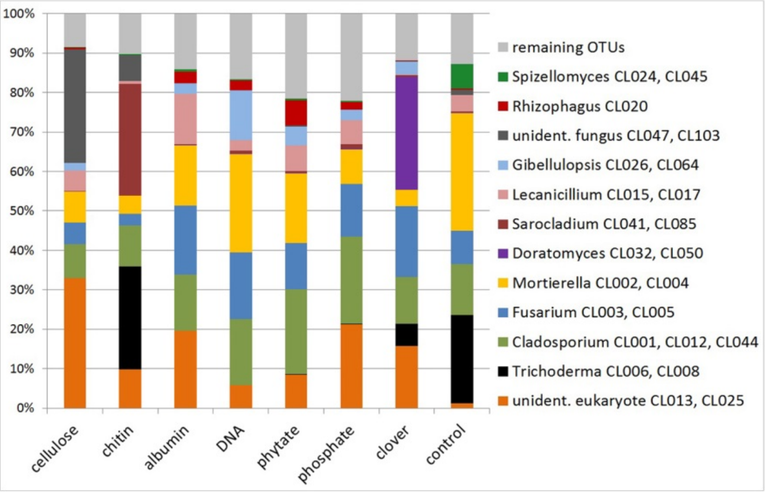

Figure S7 | Abundance of ammonia oxidizers as assessed with the specific CTO primers (**A**) and the abundance of *AmoA* gene (**C**) in the soil of different system compartments as detected with previously described primers (see Supplementary Table S1 for details) and the correlation between the abundance of these molecular abundance data and the developmental response of arbuscular mycorrhizal (AM) hyphae to the different soil amendments in root-free patches (**B** and **D**, respectively) assessed by quantitative real-time PCR with AM taxa-specific markers. Black bars, closed circles, and dashed regression lines refer to pots inoculated with *Rhizophagus irregularis,* whereas empty bars, open symbols, and dotted regression lines refer to pots inoculated with *Claroideoglomus claroideum*. Bars represent means (n = 4) with associated standard errors. Solid regression lines show the correlations of all data pooled across the two inoculation treatments. All plotted correlations were significant at the level of p < 0.05 or lower.

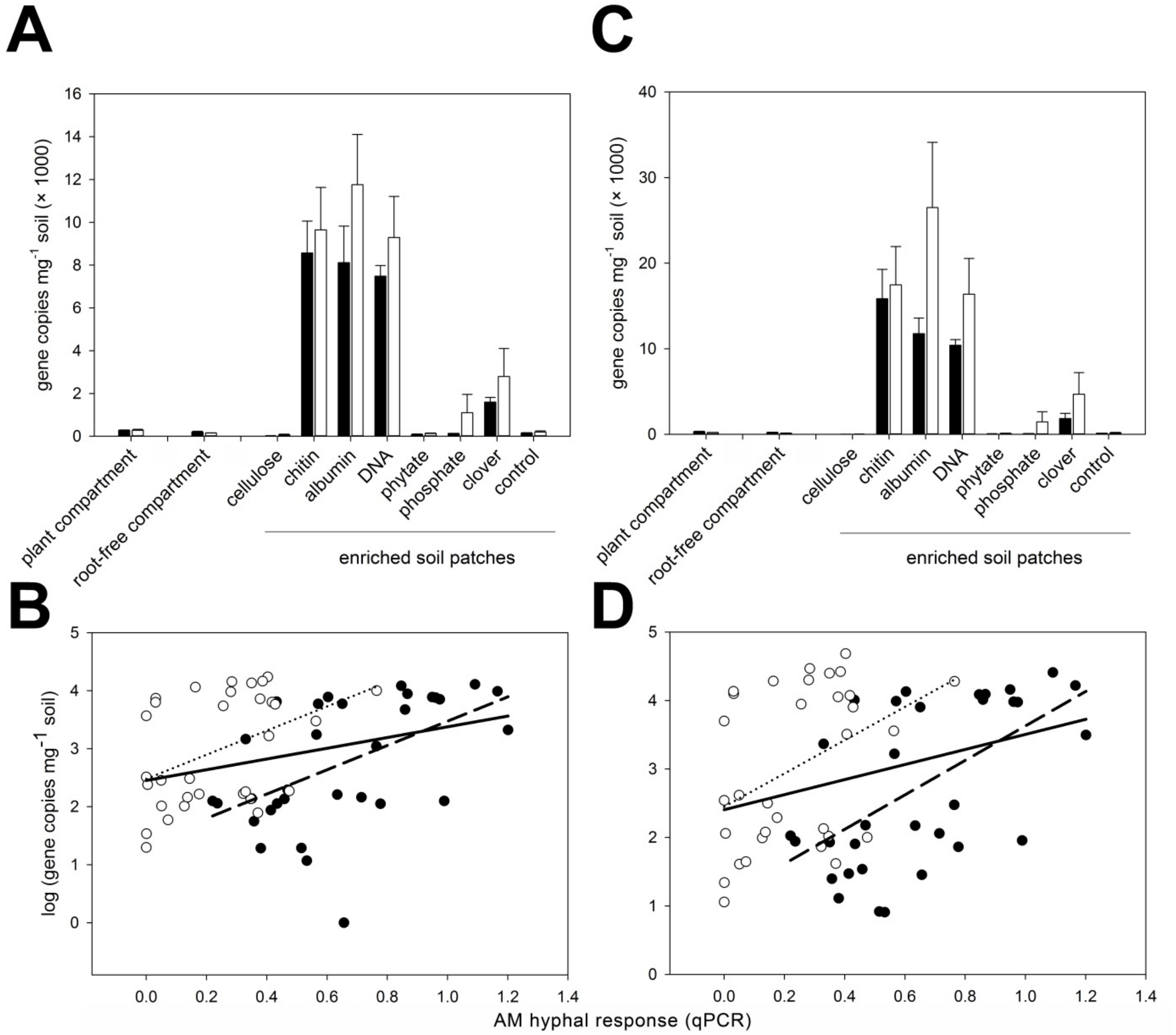

Figure S8 | Isotopic composition of nitrogen (N) in the shoots and roots of *Medicago truncatula* plants as affected by the identity of arbuscular mycorrhizal fungus and time of harvest. Delta ^{15}N values are shown indicating isotopic composition of the samples relative to the international standard (aerial N_2). Black bars – plants colonized by *Rhizophagus irregularis*, white bars – plants colonized by *Claroideoglomus claroideum*. Shown are means and standard errors (*n* = 4).

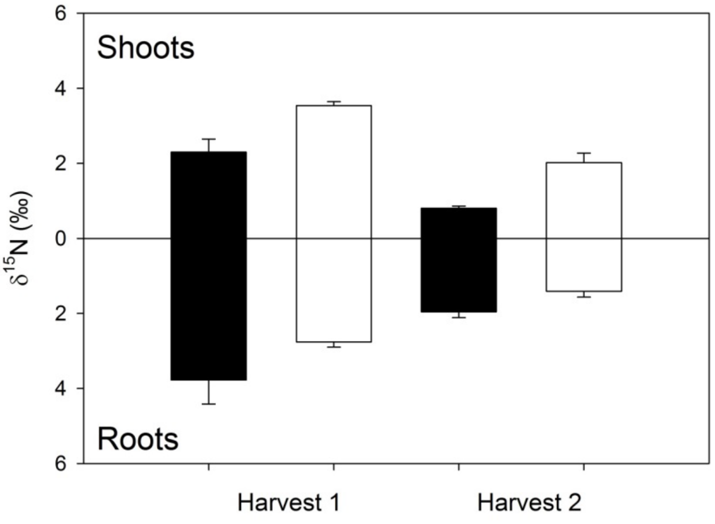

Supplement: Supplementary file 1 [file Presentation_1.PDF]
